# Psychological Impact of Type 1 Diabetes Mellitus on Parents of Children With Diabetes

**DOI:** 10.7759/cureus.99767

**Published:** 2025-12-21

**Authors:** Aida S Al Jabri, Hussain A Al Ghadeer, Afrah A Alshammari, Sabah A Alshuwish, Eyad A Bohassan, Rehab R Alhamad, Abdulhameed A Albunyan, Eman F Alamer

**Affiliations:** 1 Paediatrics, Almoosa Health Group, Al Ahsa, SAU; 2 Paediatrics, Maternity and Children Hospital, Al Ahsa, SAU; 3 Paediatrics, Ministry of National Guard Health Affairs, Al Ahsa, SAU; 4 Nursing, Ministry of National Guard Health Affairs, Al Ahsa, SAU; 5 Paediatrics, Imam Abdulrahman Bin Faisal University, Dammam, SAU

**Keywords:** alahsa, anxiety, depression, diabetes mellitus, psychological impact, quality of life, saudi arabia, stress

## Abstract

Introduction

Type 1 diabetes mellitus (T1DM) is a chronic condition with a rising incidence worldwide in both developed and developing countries. A chronic and progressive disease, diabetes is a challenge for children, adolescents, and their parents because they need special support to keep it under control. A better understanding of the relationship of parental psychological responses to having a child with T1DM is necessary to guide clinical practice and future research.

Aim

The aim of this study is to assess the psychological well-being and quality of life of parents of children with T1DM and to examine the factors associated with parental psychological distress.

Methodology

A multicenter, cross-sectional descriptive study was conducted on children (≤14 years) with T1DM followed in endocrinology clinics across Saudi Arabia from January 2022 to December 2024. Parents completed the Pediatric Quality of Life Inventory (PedsQL) and the Depression, Anxiety, and Stress Scale - 21 (DASS-21). Data were analyzed using descriptive statistics and comparative tests to evaluate associations between psychological distress and relevant factors.

Results

A total of 208 parents of children with T1DM participated in the study. The mean overall quality-of-life score was 43.7 ± 21.3, indicating a moderate impact on parental well-being. The mean DASS-21 score was 23.5 ± 16.9, with stress showing the highest subscale score (9.2 ± 5.9). Longer duration of the child’s diabetes and father’s age were significantly associated with higher parental DASS-21 scores (p = 0.032 and p = 0.023, respectively).

Conclusion

Parents of children with T1DM experience notable psychological distress and reduced quality of life. Longer disease duration and certain parental characteristics are associated with higher distress levels. These findings highlight the need for psychosocial support, early screening, and targeted interventions to support families managing childhood diabetes.

## Introduction

Type 1 diabetes mellitus (T1DM) is one of the most common chronic autoimmune diseases, characterized by the destruction of pancreatic β-cells and the resulting inability to produce insulin [1]. Clinically, children typically present with the classic symptoms of hyperglycemia, including polyuria, polydipsia, polyphagia, weight loss, and, in more severe cases, fatigue, dehydration, or diabetic ketoacidosis (DKA) at initial diagnosis [1]. During the last several decades, the prevalence and incidence of T1DM are increasing worldwide, representing a major public health concern that the number of diabetic patients has steadily increased. In 2019, according to the International Diabetes Federation’s (IDF) Diabetes Atlas (9th edition), more than a million children worldwide have T1DM [2]. In 2015, the IDF estimated the prevalence in Saudi Arabia (KSA) to be 16,100 diabetic patients (0-14 years), accounting for one-quarter of total diabetics in the Middle East and North Africa region (MENA) [3]. In 2017, the IDF reported that KSA had the highest number of T1DM in MENA, with more than 35,000 cases (0-19 years) and the highest number of newly diagnosed cases (3,900) [2].

Glycaemic control in patients with T1DM can be achieved by regular insulin therapy through continuous subcutaneous insulin infusion (CSII) or insulin pump therapy. To achieve the targeted level of adherence, regular blood glucose monitoring, follow-up, and adaptation to it as a lifelong disease are necessary. T1DM is a progressive and chronic autoimmune condition in which the destruction of pancreatic β-cells continues over time, leading to an ongoing decline in endogenous insulin production. As insulin deficiency worsens, patients and families must adapt to increasing management demands, including frequent glucose monitoring, dose adjustments, complication surveillance, and appropriate use of diabetes technology [4]. All of these aspects contribute to psychological impacts on parents and poor quality of life (QOL), as well as to children not being able to live a normal, healthy life [5]. Having a child with T1DM is considered an overwhelming experience that needs constant monitoring. Parental burden increases through constant worrying, anxiety, depression, and stress in efforts to promote children’s optimal life, growth, and development [6]. The estimated prevalence of psychological distress among parents of children with T1DM ranges from 20% to 30%, reflecting the substantial emotional and caregiving burden associated with managing a chronic pediatric condition [7]. Parents reported clinically significant distress and manifestations of anxiety, depression, and/or posttraumatic stress disorder [8,9]. Adolescents with T1DM are at least at double the risk of developing depression and anxiety disorders [10-12]. In addition, the psychological impact influences glycaemic control. As a result, the burden on patients, parents, health care, and KSA increases [13-17].

The psychological impact on parents and family life is different depending on whether it involves depression or anxiety. Depressed parents suffer an unsteady life, lower parental involvement and warmth, and family conflict [18,19]. Thus, parental psychological distress has a negative impact on parenting and family life. Health-related quality of life (HRQOL) reflects the impact of health status on an individual’s daily activities, functioning, and overall well-being, capturing physical, emotional, and social domains [20,21]. HRQOL is closely linked to T1DM, as metabolic control and disease burden influence daily functioning and psychosocial outcomes [21]. Importantly, HRQOL can also be assessed in parents and caregivers, as chronic pediatric diseases affect family functioning, stress, emotional well-being, and daily life demands [22]. Therefore, monitoring QOL in clinical practice is essential for identifying high-risk patients and applying early intervention. The objective of this study was to assess the psychological well-being and HRQOL of parents of children with T1DM, and to examine the factors associated with parental psychological distress.

## Materials and methods

Study area/setting

A cross-sectional, multicenter descriptive study was conducted on parents of children (≤14 years) with T1DM attending endocrinology clinics in KSA between January 2022 and December 2024. A convenience sampling technique was used to recruit eligible participants during routine clinic visits. The study included parents of children diagnosed with T1DM for at least one year, receiving daily insulin therapy, and without other chronic comorbidities. Parents were eligible if they provided written informed consent. Children were excluded if they had additional chronic illnesses, congenital disorders, psychiatric conditions, or were receiving medications that could influence glycemic control, such as systemic corticosteroids, high-dose topical steroids, or growth hormone therapy. Ethical approval for this study was obtained from the appropriate institutional review board in Maternity and Children Hospital in Al Ahsa (approval number: 1003-EP-2024), and the study was conducted in accordance with the principles of the Declaration of Helsinki.

Data collection

Data were collected using a predesigned questionnaire distributed to parents or caregivers online or through in-person interviews. The questionnaire included items relevant to the study objectives and captured sociodemographic information such as the child’s age, gender, duration of diabetes, most recent glycated hemoglobin (HbA1c) level, parents’ education levels, family income, household size, and number of children. HbA1c values were parent-reported based on the latest documented measurement from the child’s routine diabetes follow-up visit; no independent laboratory testing was performed by the study team.

Assessment of parent's QOL

Parental QOL was assessed using the PedsQL™ Family Impact Module, a validated instrument designed to measure the effect of pediatric chronic illness on parents and family functioning. The scale assesses parental physical, emotional, social, and cognitive functioning, as well as communication and worry. Responses are scored on a 5-point Likert scale (0 = never, 1 = almost never, 2 = sometimes, 3 = often, 4 = almost always) and linearly transformed to a 0-100 score, with lower scores reflecting poorer QOL. The instrument demonstrates strong reliability and validity, with Cronbach’s alpha values consistently >0.80 in pediatric chronic disease research [22].

Pediatric Quality of Life Inventory (PedsQL™) Diabetes Module

The PedsQL™ Diabetes Module was used to assess diabetes-specific QOL in children aged 2-18 years. The module includes 28 items across five subscales: diabetes symptoms (11 items, score range 0-44), treatment barriers (4 items, 0-16), treatment adherence (7 items, 0-28), worry (3 items, 0-12), and communication (3 items, 0-12). Each item is scored on a 5-point Likert scale (0 = never to 4 = almost always) and linearly transformed. Higher scores indicate a better QOL. The instrument has established reliability and validity in pediatric diabetes populations.

PedsQL™ Generic Core Scale (child self-report)

Child's general QOL was assessed using the PedsQL™ Generic Core Scale. The child self-report includes 23 items across four domains: physical functioning (8 items, 0-32), emotional functioning (5 items, 0-20), social functioning (5 items, 0-20), and school functioning (5 items, 0-20). Items are scored on the same 5-point Likert scale and linearly transformed to a 0-100 scale, where higher scores reflect a better QOL. Self-report was ensured by having children complete the questionnaire independently when age-appropriate (≥8 years), with parental assistance only for reading clarification without influencing responses.

Depression, Anxiety, and Stress Scale (DASS-21)

Parental psychological distress was measured using the validated DASS-21, which contains 21 items divided into three subscales: depression, anxiety, and stress (seven items each). Each item is rated from 0 to 3, with higher scores indicating more severe symptoms. Subscale scores are summed and multiplied by two according to the recommended scoring method. The DASS-21 has demonstrated good psychometric validity in assessing psychological distress in adults [23].

Statistical analysis

Data were coded and analyzed using IBM SPSS Statistics for Windows, Version 26 (Released 2018; IBM Corp., Armonk, New York, United States). Descriptive statistics (means, standard deviations, frequencies, and percentages) were used to summarize sociodemographic and clinical characteristics. Independent samples t-tests and one-way analysis of variance (ANOVA) were used to compare mean scale scores across groups, while chi-square tests were applied for categorical variables. Pearson correlation coefficients were calculated to examine associations between continuous variables such as duration of diabetes, parental age, and psychological distress scores. A p-value < 0.05 was considered statistically significant.

## Results

A total of 208 parents participated in this study, representing 208 children diagnosed with T1DM. Table 1 clarifies the sociodemographic characteristics of the parents and their children. Over half of the parents (113, 54.3%) came from private hospitals, while 95 (45.7%) were from public hospitals. Of all parents, 109 (52.4%) were women and 99 (47.6%) were men. Mothers were the main caregivers in 179 cases (86.1%), while fathers constituted 27 cases (13%). Most parents (194, 93.3%) reported no additional health issues, while 14 (6.7%) had conditions such as thyroid disease, celiac disease, or asthma. Regarding the children, 82 (39.4%) were diagnosed less than two years ago, 69 (33.2%) between two and four years ago, and 57 (27.4%) had been living with T1DM for five to 10 years. Hospital admissions for serious events such as severe hypoglycemia or DKA were less frequent, occurring in 87 children (41.8%) during the past year. Multiple daily insulin injections were used by 183 children (88%), whereas 25 (12%) used an insulin pump. Most mothers (147, 70.7%) and fathers (130, 62.5%) were aged 31-45 years. Regarding education, 96 mothers (46.2%) and 78 fathers (37.5%) held university degrees. A total of 51 families (24.5%) reported a monthly income of 3,500-6,000 Saudi Riyal (SR), while 50 families (24%) reported an income of 10,000-15,000 SR. Families typically had four to five members (80, 38.5%), and most had two to three children (77, 37%).

**Table 1:** Sociodemographic profile of the participants K/C: known case; DM: diabetes mellitus; DKA: diabetic ketoacidosis

Sociodemographic variable	Categories	Frequency (%)
Setting	Private hospital	113 (54.3)
	Public hospital	95 (45.7)
Gender	Male	99 (47.6)
	Female	109 (52.4)
Relationship to the child	Father	55 (26.4)
	Mother	150 (72.1)
	Sister	3 (1.4)
Taking care	Father	27 (13)
	Mother	179 (86.1)
	Sister	2 (1)
K/C of other disease	No	194 (93.3)
	Yes	14 (6.7)
If yes, what?	Thyroid	3 (21.4)
	Celiac	3 (21.4)
	Seizure	1 (7.1)
	Deafness	1 (7.1)
	Autism	4 (28.6)
	G6PD	1 (7.1)
	Asthma	1 (7.1)
Diagnosis of DM since when	Less than 2 years	82 (39.4)
	2 to 4 years	69 (33.2)
	5 to 10 years	57 (27.4)
Severe hypoglycemia led to admission per year	No	118 (56.7)
	Once	57 (27.4)
	2 to 4 times	26 (12.5)
	5 to 10 Times	7 (3.4)
DKA per year	No	118 (56.7)
	Once	57 (27.4)
	2 to 4 times	26 (12.5)
	5 to 10 times	7 (3.4)
Insulin received via	Insulin pen	183 (88)
	Insulin pump	25 (12)
Mother age	17 to 30 years	34 (16.3)
	31 to 45 years	147 (70.7)
	more than 45	27 (13)
Father age	17 to 30 years	7 (3.4)
	31 to 45 years	130 (62.5)
	more than 45	71 (34.1)
Mother education	Illiterate	6 (2.9)
	Primary school	11 (5.3)
	Intermediate school	24 (11.5)
	Secondary school	66 (31.7)
	University	96 (46.2)
	Postgraduate	5 (2.4)
Father education	Illiterate	3 (1.4)
	Primary school	11 (5.3)
	Intermediate school	26 (12.5)
	Secondary school	77 (37)
	University	78 (37.5)
	Postgraduate	13 (6.3)
Family income	Less than 3500 SR	21 (10.1)
	3500 to 6000 SR	51 (24.5)
	6000 to 10000 SR	49 (23.6)
	10000 to 15000 SR	50 (24)
	More than 15000 SR	37 (17.8)
Number of family members	Three	9 (4.3)
	4 to 5 members	80 (38.5)
	More than 5	119 (57.2)
Number of children in the family	One	24 (11.5)
	2 to 3	77 (37)
	4 to 5	58 (27.9)
	More than 5	49 (23.6)

Table 2 presents the sociodemographic profile for age and HbA1c levels among the study participants. The children’s ages ranged from two to 14 years, with an average age of 10.04 ± 3.81 years. Their HbA1c levels ranged between 6% and 17%, with a mean of 9.26 ± 2.1%.

**Table 2:** Sociodemographic profile by age and HbA1c HbA1c: glycated hemoglobin

Sociodemographic variable	Min	Max	Mean ± SD
Age	2	14	10.04 ± 3.81
HbA1c	6	17	9.26 ± 2.138

Table 3 presents the HRQOL scores of parents of children with T1DM based on the PedsQL™ Family Impact Module. The overall parental QOL score showed a mean of 43.7 ± 21.3, indicating a moderate level of impact, where lower scores reflect poorer QOL. For the domain-specific scores, the parent HRQOL summary score (diabetes-related functioning) had a mean of 17.3 ± 8.5. The two treatment-related subscales, treatment barriers and treatment adherence, had mean scores of 6.1 ± 3.8 and 9.5 ± 7.8, respectively. The worry domain showed a mean score of 5.6 ± 3.7, while communication had a mean of 5.2 ± 3.9. These findings suggest that parents experience notable challenges, particularly in treatment demands, worry, and communication related to their child’s diabetes.

**Table 3:** Quality of life (QOL) among parents of children with type 1 diabetes mellitus (DM)

QOL	Min	Max	Mean ± SD
Diabetes	0.00	44	17.3029 ± 8.49038
Treatment Barriers	0.00	16	6.0913 ± 3.80647
Treatment Adherence	0.00	28	9.4856 ± 7.79445
Worry	0.00	12	5.625 ± 3.7318
Communication	0.00	12	5.1971 ± 3.86919
Quality of Life	0.00	112	43.7019 ± 21.2666

Table 4 shows scores for the psychological well-being of parents of children with T1DM. The mean anxiety score is 7.1 ± 5.7 out of 21. The depression score has a slightly higher mean of 7.3 ± 6.1 out of 21. Stress levels are notably higher, with a mean score of 9.2 ± 5.9 out of 21. The total DASS-21 score had a mean of 23.5 ± 16.9.

**Table 4:** Depression, Anxiety, and Stress Scale - 21 items (DASS-21)

DASS	Min	Max	Mean ± SD
Anxiety	0.00	21	7.1010 ± 5.72176
Depression	0.00	21	7.2548 ± 6.11859
Stress	0.00	21	9.1827 ± 5.94949
DASS-21	0.00	63	23.5385 ± 16.91863

Table 5 presents the results of the Generic Core Scale of the Pediatric QOL questionnaire. The scale assesses children’s perceived QOL across various domains. On average, children in the study reported moderate levels of functioning in all domains, with scores ranging from 3.28 (social functioning) to 7.72 (physical functioning) out of a possible maximum score of 15 or 24, depending on the domain.

**Table 5:** Pediatric Quality of Life Inventory (PedsQL) - generic core scale

Generic Core Scale of PedsQL	Min	Max	Mean ± SD
Physical Functioning	0.00	24	7.7212 ± 5.69169
Emotional Functioning	0.00	37	7.1538 ± 4.3099
Social Functioning	0.00	15	3.2837 ± 3.7276
School Functioning	0.00	15	4.9038 ± 4.17496
Generic Core Scale of PedsQL	0.00	66	23.0625 ± 13.17372

Table 6 shows the relationship between different factors and diabetic child parents psychological well-being. One significant finding is that the years of diagnosis of diabetes are linked to higher DASS-21 scores. Children diagnosed for five to 10 years had predominantly higher scores (49.78 ± 24.97) compared to those diagnosed for less than two years (40.42 ± 19.14) and two to four years (42.56 ± 19.53), with a p-value of 0.032. Additionally, the father’s age also showed a significant relationship with DASS-21 scores. Fathers aged 31 to 45 years had higher scores (46.8 ± 20.12) than those aged 17 to 30 years (36.42 ± 14.7) and those over 45 years (38.73 ± 22.89), with a p-value of 0.023. Other factors such as gender, setting, relationship to the child, presence of other chronic diseases, frequency of hypoglycemia, and educational levels did not show significant differences in DASS-21 scores (p-values were all higher than 0.05).

**Table 6:** Relationship between demographic characteristics and DASS-21 scores K/C: known case; DKA: diabetic ketoacidosis; HbA1c: glycated hemoglobin; DASS-21: Depression, Anxiety and Stress Scale - 21

Characteristics	Value	P-value
Setting		0.27
Public	42.21 ± 21	
Private	45.47 ± 20.96	
Gender		0.93
Male	43.83 ± 21.73	
Female	43.57 ± 20.93	
Relationship		0.337
Father	40.35 ± 20.47	
Mother	45 ± 21.64	
Sister	38 ± 10.1	
Taking care		0.525
Father	47.88 ± 19.7	
Mother	43.12 ± 21.57	
Sister	38.5 ± 9.19	
K/C of other chronic disease		0.895
No	43.64 ± 21.56	
Yes	44.42 ± 17.14	
Years of diagnosis		0.032
Less than 2 years	40.42 ± 19.14	
2 to 4 years	42.56 ± 19.53	
5 to 10 years	49.78 ± 24.97	
Age	10.04 ± 3.81	0.13
Hypoglycemia		0.11
Once	44 ± 19.3	
2-4 times	51.69 ± 21.74	
DKA		0.11
Once	44.017 ± 19.3	
2 to 4 times	51.69 ± 21.74	
Insulin received via		0.956
Insulin pen	43.73 ± 21.739	
Insulin pump	43.48 ± 17.79	
HbA1c	43.7 ± 21	0.652
Mother age		0.292
17 to 30 years	47.17 ± 22.6	
31 to 45 years	43.83 ± 21.39	
More than 45	38.59 ± 18.33	
Father age		0.023
17 to 30 years	36.42 ± 14.7	
31 to 45 years	46.8 ± 20.12	
More than 45 years	38.73 ± 22.89	
Mother’s Education		0.175
Illiterate	32.83 ± 23.54	
Primary school	48 ± 32.77	
Intermediate school	45.41 ± 22.41	
Secondary school	44.757 ± 19.75	
University	41.68 ± 19.499	
Postgraduate	63.8 ± 29.98	
Father’s Education		0.909
Illiterate	46.33 ± 29	
Primary school	40.18 ± 22.96	
Intermediate school	40.07 ± 17.49	
Secondary school	43.81 ± 23.83	
University	45.29 ± 19.86	
Postgraduate	43.07 ± 19.94	
Family income		0.432
Less than 3500 SR	42.8 ± 20.52	
3500 to 6000 SR	48 ±24.95	
6000 to 10,000	40.61 ± 19.41	
10.000 to 15.000	44.68 ± 19.31	
More than 15,000	41 ± 21	
Number of family members		0.97
Three	43.77 ± 12.3	
4 to 5 members	43.27 ± 19.5	
More than 5	43.98 ± 22.97	
Number of children		0.338
One	44.66 ± 20.18	
2 to 3	44.46 ± 19.23	
4 to 5	46.25 ± 23.27	
More than 5	39 ± 22.23	

## Discussion

In this study, we found that mothers mainly take on the caregiving role, which matches traditional expectations. Most parents reported that they had no other health problems, focusing mainly on managing their child’s diabetes. The different lengths of time since diagnosis suggest that parents have varying levels of experience, which may affect how they manage their child’s condition. The low incidence of severe hospital admissions suggests effective diabetes management. Pen injectors are the preferred method for insulin administration, likely due to their convenience. The majority of parents are in the 31- to 45-year age range and hold university degrees, which may contribute to better diabetes management practices. The demographic data of the children reveal a wide age range and HbA1c levels. The average levels suggest that while some children have good glycemic control, others require improved management strategies. These findings are consistent with previous studies that emphasize the burden of diabetes management on families and the importance of education and support in achieving better health outcomes. 

The main objective of this study was to assess the psychological well-being and QOL of parents of children with T1DM. The findings indicate that the parents’ overall QOL is considerably affected by their child’s condition. The study reflects the multifaceted challenges these parents face, including the stress of managing diabetes, the burden of treatment, and the constant worry about their child’s health. In more detail, the diabetes management score highlights the significant impact of managing the daily demands of the condition. The scores for the treatment barriers and treatment adherence domains suggest different levels of difficulty associated with diabetes care, where lower scores reflect greater burden and more challenges in adhering to treatment requirements. The worry category highlighted the persistent anxiety parents experience regarding their child’s health and long-term outcomes. Communication difficulties further suggest that parents may struggle with discussing and coordinating aspects of their child’s diabetes management effectively. The worry category highlighted the inescapable anxiety parents experience. In addition, communication difficulties suggest that parents may struggle with discussing and managing their child’s diabetes effectively. These findings align with previous research by Faulkner and Clark [24] that underlines the considerable emotional and practical challenges in managing a child’s diabetes. The referenced study also noted that parental life satisfaction is significantly impacted by the burden of the child’s diabetes and the frequency of discussing it with others. Other studies highlight the substantial emotional and practical challenges faced by parents of children with T1DM. The referenced studies showed that parents’ experiences revealed an intense emotional burden and practical difficulties similar to those observed in our study [25]. Additionally, the study on parental QOL noted that the child’s condition impacts family life and parental well-being, consistent with our findings [26,27]. All studies underline the importance of comprehensive support systems, effective diabetes education, and tailored interventions to improve these parents’ QOL and psychological well-being [7].

Regarding psychological well-being, the study findings reflect moderate levels of anxiety and depression, consistent with findings from other studies [7]. Whittemore et al. [7] showed that the distress levels ranged from 10% to 74%, with an average of 33.5% at diagnosis and 19% after one to four years. Parental psychological distress was linked to higher child-reported stress and depressive symptoms, problematic behavior, and lower QOL. It also negatively affected diabetes management. The authors found that parents perceived T1DM as a disruptive diagnosis, causing significant family disruption. Although adjustment occurred over time, ongoing stress was reported [28]. Comparing these findings with our study, both studies emphasize the significant psychological burden on parents of children with T1DM. Our study also reported moderate levels of anxiety, depression, and stress among parents, reflecting the emotional challenges highlighted in the referenced study. These findings were also concurrent with many other studies where higher parenting stress and lower parenting competence, self-efficacy, and parenting satisfaction were demonstrated in parents of children with T1DM [29-33]. These findings emphasize the need for comprehensive support systems for parents of children with T1DM. Psychological support and targeted interventions are crucial to help parents manage the emotional and psychological challenges associated with their child’s condition. Effective diabetes education, counselling, and support groups could play a vital role in improving these parents’ QOL and psychological well-being.

Strengths and limitations

This study has several strengths, including its multicenter design, relatively large sample size, and the use of validated instruments (PedsQL™ and DASS-21) to assess parental QOL and psychological well-being. It provides important insight into the psychosocial burden experienced by parents of children with T1DM in KSA, a topic that remains under-studied in the region. However, the study has limitations. Its cross-sectional design prevents establishing causality, and reliance on parent-reported HbA1c values may introduce recall or reporting bias. The study did not assess child behavioral factors or parental coping mechanisms, which may influence parental stress and QOL. Additionally, the use of convenience sampling may limit generalizability to all T1DM families.

Implications

The findings highlight the substantial psychological and QOL challenges faced by parents caring for a child with T1DM. This underscores the need for routine psychosocial screening in diabetes clinics, family-centered education programs, and accessible psychological support services to improve overall disease management and family well-being.

Future directions

Future research should consider longitudinal designs to track changes in parental psychological outcomes over time and evaluate the effect of structured support programs. Studies incorporating objective clinical markers, child behavioral data, and qualitative exploration of parental experiences would provide a more comprehensive understanding of factors influencing parental well-being.

## Conclusions

Our study revealed significant emotional and psychological challenges that parents of children with T1DM face. Moderate levels of anxiety, depression, and stress were prevalent among parents, reflecting the substantial burden of managing the condition. These findings align with other research, underscoring the impact of parental psychological distress on both the child’s well-being and diabetes management. The study stressed the need for comprehensive support systems, effective diabetes education, and targeted interventions. Providing psychological support, counselling, and establishing support groups for parents can improve their QOL and psychological well-being. Additionally, tailored educational programs to enhance diabetes management skills and communication can help mitigate the emotional strain. Future research should focus on developing and evaluating interventions that address these challenges to support parents in managing their child’s T1DM more effectively.
